# Acid ceramidase expression and biomarker potential in patients with locally advanced rectal cancer

**DOI:** 10.17305/bb.2025.13275

**Published:** 2025-11-28

**Authors:** Jasna Bjelanovic, Katarina Zeljic, Marko Miladinov, Goran Barisic, Sandra Dragicevic

**Affiliations:** 1 Center for Medical Biochemistry, University Clinical Center of Serbia, Belgrade, Serbia; 2 Faculty of Biology, University of Belgrade, Belgrade, Serbia; 3 Clinic for Digestive Surgery-First Surgical Clinic, University Clinical Center of Serbia, Belgrade, Serbia; 4 Faculty of Medicine, University of Belgrade, Belgrade, Serbia; 5 Gene Regulation in Cancer Group, Institute of Molecular Genetics and Genetic Engineering, University of Belgrade, Belgrade, Serbia

**Keywords:** Acid ceramidase, *ASAH1* gene, CALLY index, neoadjuvant chemoradiotherapy, rectal cancer

## Abstract

Acid ceramidase (AC), a pivotal enzyme in sphingolipid metabolism, has been associated with various cancers; however, its specific role in rectal cancer remains poorly understood. This study aimed to explore the clinical significance of AC gene and protein expression in rectal cancer. We analyzed the expression of *ASAH1*, *BAX*, and *BCL2* through quantitative Real-Time PCR in paired tumor and non-tumor tissue samples obtained from patients with locally advanced rectal cancer (LARC) prior to neoadjuvant chemoradiotherapy. Additionally, serum AC levels and standard biochemical parameters were assessed. We further evaluated *ASAH1* expression using RNA-seq data from publicly available TCGA-READ datasets accessed via the UCSC Xena Browser. Two approaches indicated a significant reduction in *ASAH1* expression in tumor tissue (*P* ═ 0.004 and *P* < 0.001, respectively). Receiver operating characteristic curve analysis revealed a modest capacity for *ASAH1* expression to differentiate between tumor and non-tumor tissue in LARC patients (AUC = 0.652, *P* ═ 0.042). No correlation was observed between *ASAH1* expression and the *BAX/BCL2* ratio in tumor tissue, nor with serum AC levels or the CRP-albumin-lymphocyte (CALLY) index. Conversely, serum AC levels exhibited a negative correlation with the *BAX/BCL2* ratio (rs ═ −0.536, *P* ═ 0.002, FDR-adjusted *q* ═ 0.021). Furthermore, *ASAH1* expression, AC levels, and the CALLY index were not linked to overall survival or treatment response. A key finding of this study is the inverse relationship between serum AC levels and the pro-apoptotic status of tumor tissue, suggesting that circulating AC may provide valuable insights into tumor apoptotic activity. Further large-scale studies are necessary to validate these preliminary findings and elucidate the biomarker potential of AC in rectal cancer.

## Introduction

Colorectal cancer (CRC) presents a significant global health challenge, ranking third in incidence and second in cancer-related mortality, according to GLOBOCAN 2022 data [1]. In the United States, it is estimated that 46,000 of the 153,000 new CRC cases in 2023 are rectal cancers, representing approximately 30% of all CRC cases [2]. Among these, nearly 45% are classified as locally advanced rectal cancer (LARC), defined as stage II and III disease [3]. While early-stage rectal cancer is often managed with surgery alone, LARC typically necessitates a multimodal treatment approach. Advanced stages are commonly treated with neoadjuvant radiotherapy and/or chemotherapy to reduce tumor size prior to surgical resection [4]. However, the response to neoadjuvant therapy is highly variable, with only about 20% of patients achieving a complete pathological response [5]. The clinical trajectory of CRC varies based on tumor location, with rectal cancer generally associated with poorer relapse-free survival compared to colon cancer [6]. Furthermore, systemic inflammation significantly contributes to cancer progression and therapy resistance, complicating treatment outcomes [7].

Current tumor markers, such as carcinoembryonic antigen (CEA) and carbohydrate antigen 19-9 (CA 19-9), are primarily utilized for monitoring disease progression and recurrence in CRC patients. However, their limited specificity and sensitivity diminish their overall clinical utility [8]. Given the constraints of these predictive markers, there is an urgent need for more reliable and specific biomarkers to guide treatment decisions and enhance patient outcomes.

Recent years have seen growing interest in the role of sphingolipids as potential biomarkers in CRC, owing to their involvement in tumor growth, therapy resistance, and immune modulation [5, 9, 10]. Notably, recent research indicates significant differences in sphingolipid profiles based on tumor location, revealing markedly lower levels of long-chain ceramides in rectal tissue compared to colon tissue [11]. These findings suggest a potential association between tumor location and sphingolipid metabolism, underscoring the necessity for targeted biomarker studies in rectal cancer. Given the interplay between inflammation and sphingolipid metabolism, identifying specific biomarkers that reflect both tumor biology and systemic immune responses could enhance patient stratification, determining those more or less likely to respond to therapy.

Among sphingolipid molecules, ceramides are crucial in cancer as they regulate essential cellular processes such as migration, adhesion, proliferation, differentiation, growth inhibition, and apoptosis [9, 12]. Although ceramides act as tumor suppressors, their metabolism is frequently altered in cancer cells, contributing to therapy resistance and disease progression [5, 9]. This imbalance may arise not only from disrupted *de novo* synthesis but also from increased degradation mediated by acid ceramidase (AC), an enzyme encoded by the N–acylsphingosine amidohydrolase (*ASAH1*) gene. AC catalyzes the breakdown of ceramides into sphingosine, which is a precursor for sphingosine-1-phosphate (S1P) [5]. S1P promotes cell survival, proliferation, and resistance to therapy, fostering a pro-tumorigenic environment [5, 9]. Tumors often shift sphingolipid metabolism toward S1P with elevated AC activity, which contributes to systemic inflammation that supports tumor growth [13, 14]. In this context, AC represents a promising target for novel therapeutic strategies [13, 15, 16]. However, studies specifically investigating AC in rectal cancer remain limited [17–19]. A recent study utilizing CRC cell lines and rectal cancer organoids confirmed the role of AC in modulating radiosensitivity, further emphasizing its relevance in the response to neoadjuvant chemoradiotherapy (nCRT) [18, 19]. Given the critical role of AC in sphingolipid metabolism and its potential influence on cancer progression, this study aimed to assess the expression patterns of AC at both the gene and protein levels in rectal cancer and to explore their clinical relevance as biomarkers.

## Materials and methods

### Patients

This study involved 30 patients diagnosed with LARC, aged 34–83 years (63.3% male), who were recruited from the Clinic for Digestive Surgery - First Surgical Clinic at the University Clinical Center of Serbia between April 2019 and February 2022. Inclusion criteria were histologically confirmed rectal adenocarcinoma, no prior preoperative treatment, absence of distant metastases (M0 status), and signed informed consent. Patients who opted not to provide informed consent were excluded from the study. The initial clinical stage was determined using endoscopic ultrasound and pelvic magnetic resonance imaging, while the presence or absence of metastases was assessed through computed tomography and/or magnetic resonance imaging.

At diagnosis, biopsy samples of the primary tumor and adjacent healthy mucosa were collected from each patient, promptly processed, and stored at --80 ^∘^C for subsequent RNA extraction and gene expression analysis. Blood samples were also obtained for standard hematological and biochemical analyses, with serum aliquots stored at --80 ^∘^C for later quantification of AC.

Approximately five weeks post-diagnosis, patients underwent clinical re-evaluation prior to treatment initiation. Of the 30 patients, 26 received nCRT followed by surgical resection, while four patients were treated solely with systemic chemotherapy due to poor general health and/or newly detected secondary metastases in the lungs and/or liver. These four patients were excluded from the evaluative analysis of therapy response due to their deviation from the planned treatment protocol and their disease stage being classified as beyond locally advanced disease.

The nCRT regimen consisted of a total radiation dose of 50.4 Gy delivered in 28 fractions, combined with two or three cycles of chemotherapy (5-fluorouracil 425 mg/m^2^ and leucovorin 20 mg/m^2^). Following a period of 8–12 weeks, patients underwent surgical resection, and tumor regression grade (TRG) was assessed through histopathological analysis to evaluate the response to nCRT. Based on the pathological response, patients were stratified into three categories: good responders (*n* ═ 5), characterized by complete (TRG1) and near-complete (TRG2) tumor regression; moderate responders (*n* ═ 9), exhibiting moderate tumor regression (TRG3); and poor responders (*n* ═ 12), showing minimal tumor regression (TRG4) or no regression (TRG5).

Patient outcomes were assessed based on overall survival, with follow-up conducted until November 2024.

### Gene expression analysis by quantitative real-time PCR (qRT-PCR)

Total RNA was isolated from tissue samples using TRI Reagent Solution (Thermo Fisher Scientific, Waltham, MA, USA) in accordance with the manufacturer’s guidelines. RNA concentration and purity were evaluated by measuring absorbance at 260 nm and 280 nm using a BioSpec-nano spectrophotometer (Shimadzu Corporation, Kyoto, Japan). Only samples with a 260/280 ratio of 1.8–2.0 were included in subsequent analyses. For mRNA expression analysis of target genes, total RNA (2 µg) was reverse transcribed using the High Capacity cDNA Reverse Transcription Kit (Applied Biosystems, Foster City, CA, USA), following the manufacturer’s instructions. The reaction conditions included 10 min at 25 ^∘^C, 120 min at 37 ^∘^C, and 5 min at 85 ^∘^C.

The mRNA expression levels of the target genes (*ASAH1*, *BCL2*—an apoptosis regulator, and *BAX*—BCL2 associated X, apoptosis regulator) were quantified in triplicate using qRT-PCR with Power SYBR™ Green PCR Master Mix (Applied Biosystems, Foster City, CA, USA), with actin beta (*ACTB*) serving as the internal housekeeping gene control for normalization. No-template controls were included in all reactions to ensure the absence of contamination. Primer specificity was confirmed prior to the study, and melting curve analysis was conducted for all reactions to ensure product specificity. The stability of *ACTB* was validated across samples, affirming its suitability as the internal control. Primer sequences and product lengths are provided in Table 1.

**Table 1:** Primer sequences and lengths of gene fragments

**Gene**	**Primer sequence**	**Length of the fragment (bp)**
*ASAH1*	Forward 5′–TCCTTGATGATCGCAGAACGCC–3′ Reverse 5′–ACGGTCAGCTTGTTGAGGAC–3′	121
*BCL2*	Forward 5′–TCGCCCTGTGGATGACTGA–3′ Reverse 5′–CAGAGACAGCCAGGAGAAATC–3′	134
*BAX*	Forward 5′–TGGCAGCTGACATGTTTTCTGAC–3′ Reverse 5′–TCACCCAACCACCCTGGTCTT–3′	195
*ACTB*	Forward 5′–GGACTTCGAGCAAGAGATGG–3′ Reverse 5′–AGGAAGGAAGGCTGGAAGAG–3′	138

Abbreviations: *ASAH1*: N–acylsphingosine amidohydrolase 1; *BCL2:* BCL2 apoptosis regulator; *BAX*: BCL2 associated X, apoptosis regulator; *ACTB:* Actin beta.

qRT-PCR was performed on the 7500 Real-Time PCR System (Applied Biosystems, Foster City, CA, USA) under the following thermal cycling conditions: 2 min at 50 ^∘^C, 10 min at 95 ^∘^C, followed by 40 cycles of 15 s at 95 ^∘^C and 1 min at 60 ^∘^C. The comparative ΔCt method was utilized to calculate relative gene expression, where ΔCt equals the Ct of the target gene minus the Ct of the housekeeping gene. The expression levels of the analyzed target genes were reported as 2^--ΔCt^ and were utilized for statistical analysis.

### Analysis of publicly available sequencing data

RNA-sequencing (RNA-seq) data from publicly accessible datasets were retrieved from the UCSC Xena Browser and analyzed independently to assess *ASAH1* expression in tumor and non-tumor rectal tissues (https://xenabrowser.net; accessed on June 12, 2025). The analysis utilized the Cancer Genome Atlas Rectum Adenocarcinoma (TCGA-READ) dataset, which comprised transcriptomic data from primary rectal tumors (*n* ═ 92) and non-tumor tissues (*n* ═ 10). Only TCGA-READ samples were analyzed, as normal rectal tissues from the Genotype-Tissue Expression (GTEx) Project are not available for this tissue type. Gene expression levels were reported as log_2_(FPKM+0.001), where FPKM refers to Fragments Per Kilobase of transcript per Million mapped reads, quantified using the RSEM (RNA-Seq by Expectation-Maximization) method.

### AC measurement in serum samples

Quantification of AC levels in serum samples was conducted using a commercial Human ASAH1 ELISA kit (Assay Genie, Dublin, Ireland), following the manufacturer’s instructions. Serum samples were incubated with a biotin-conjugated monoclonal antibody specific for AC, followed by the addition of avidin-conjugated horseradish peroxidase. After thorough washing, 3,3′,5,5′-tetramethylbenzidine substrate was added, resulting in the development of a blue color. The reaction was halted by the addition of sulfuric acid, which turned the solution yellow. Absorbance was measured at 450 nm using a microplate reader, with the intensity of the colorimetric signal being directly proportional to the concentration of AC in the samples. The assay had a calibration range of 0.156–5 ng/mL, a limit of detection of 0.094 ng/mL, and a limit of quantification of 0.156 ng/mL. The intra-assay coefficient of variation was < 8%, and the inter-assay coefficient of variation was < 10%. A dilution factor of 2 was applied, and all samples were measured in duplicate in accordance with good laboratory practices.

### Ethical statement

Ethical approval for sample collection was obtained as part of the strategic project MOHERATEKA (grant number F–69) from the Ethics Committee of the Faculty of Medicine, University of Belgrade (Approval No. 1550/V–2; May 31, 2019). Although patient recruitment commenced in April 2019, sample collection and data acquisition were performed only after ethical approval was secured. The specific analyses conducted in this study received additional approval from the Ethics Committee of the University Clinical Center of Serbia (Approval No. 447/6; October 19, 2021), in accordance with the Declaration of Helsinki. Written informed consent was obtained from all participants.

### Statistical analysis

Statistical analyses were performed using the Statistical Package for Social Sciences version 20.0 (SPSS Inc., Chicago, IL, USA). Graphical representations of the results were created using GraphPad Prism version 9.0 (GraphPad Software, LLC, Boston, MA, USA). Continuous variables are presented as medians with interquartile ranges (IQR), defined as the difference between the 75th and 25th percentiles, while categorical variables are reported as numbers (percentages). The Shapiro–Wilk test indicated that the data did not follow a normal distribution; thus, appropriate non-parametric tests were employed for further analysis. Differences between independent samples were evaluated using the Mann–Whitney *U* test and Kruskal–Wallis test, while matched samples were assessed using the Wilcoxon matched-pairs signed-rank test. The degree of association between variables was calculated utilizing Spearman’s rank correlation coefficient (rs). The robustness of the correlations was further evaluated through a bootstrap procedure with 1000 resamples. Receiver operating characteristic (ROC) curve analysis and area under the curve (AUC) were employed to assess the discriminatory ability between two variables. AUC values below 0.6 indicate poor discrimination, 0.6–0.7 indicate modest discrimination, 0.7–0.8 indicate good discrimination, 0.8–0.9 indicate very good discrimination, and above 0.9 indicate excellent discriminatory power of the biomarker. Univariate Cox proportional hazards regression analysis was performed to evaluate the association between each variable and overall survival. *P* values less than 0.05 were considered statistically significant. To control for multiple testing, *P* values were adjusted using the false discovery rate (FDR) correction according to the Benjamini–Hochberg method.

## Results

This study included 30 patients diagnosed with LARC, all classified as stage III, whose demographic and clinical characteristics are outlined in Table 2. We analyzed the expression levels of the genes *ASAH1, BAX*, and *BCL2* in biopsied rectal tissue samples prior to therapy. Additionally, we assessed serum levels of AC, standard biochemical parameters, and tumor markers. The ratio of the pro-apoptotic gene *BAX* to the anti-apoptotic gene *BCL2 (BAX/BCL2*) was calculated to estimate the apoptotic status of the tumor tissue samples. Furthermore, the CALLY index was computed using the formula: albumin level (g/L) × absolute lymphocyte count (×10^9^/L) / CRP level (mg/L) × 10, to reflect nutritional, immune, and inflammatory status. The median *BAX/BCL2* ratio was 12.1 (IQR 16.0), while the median CALLY index was 2.31 (IQR 3.42), indicating variability among patients.

**Table 2:** Demographic and clinical characteristics of patients with locally advanced rectal cancer (*n* ═ 30)

**Characteristic**	**Value**
Age (years), median (IQR)	66 (13)
Males, *n* (%)	19 (63.3)
Glucose (mmol/L), median (IQR)	5.6 (1.7)
Proteins (g/L), median (IQR)	72 (4.5)
Albumin (g/L), median (IQR)	44 (2)
Cholesterol (mmol/L), median (IQR)	5.4 (1.7)
Triglycerides (mmol/L), median (IQR)	1.5 (0.6)
White blood cells (×10^9^/L), median (IQR)	7 (3)
Lymphocytes (×10^9^/L), median (IQR)	1.9 (1.1)
Neutrophils (×10^9^/L), median (IQR)	4 (2)
CRP (mg/L), median (IQR)	3.6 (5.9)
CEA (µg/L), median (IQR)	3.2 (2.9)
CA 19-9 (U/mL), median (IQR)	5.9 (13.2)
*T stage at diagnosis, n (%)*	
T2	1 (3.3)
T3	21 (70.0)
T4	8 (26.4)
*N stage at diagnosis, n (%)*	
N1	7 (23.3)
N2	23 (76.7)
*Overall stage, n (%)*	
IIIA	1 (3.3)
IIIB	7 (23.3)
IIIC	22 (73.4)
*Response to nCRT^#^, n (%)*	
TRG1	3 (11.5)
TRG2	2 (7.7)
TRG3	9 (34.6)
TRG4	11 (42.3)
TRG5	1 (3.8)
*Outcome, n (%)*	
Alive	25 (83.3)
Dead	5 (16.7)

^#^Four patients treated with chemotherapy alone were excluded from the evaluation of nCRT response. Abbreviations: IQR: Interquartile range defined as the difference between the 75th and 25th percentiles; CRP: C-reactive protein; CEA: Carcinoembryonic antigen; CA 19-9: Carbohydrate antigen 19-9; nCRT: Neoadjuvant chemoradiotherapy; TRG: Tumor regression grade.

**Figure 1.**
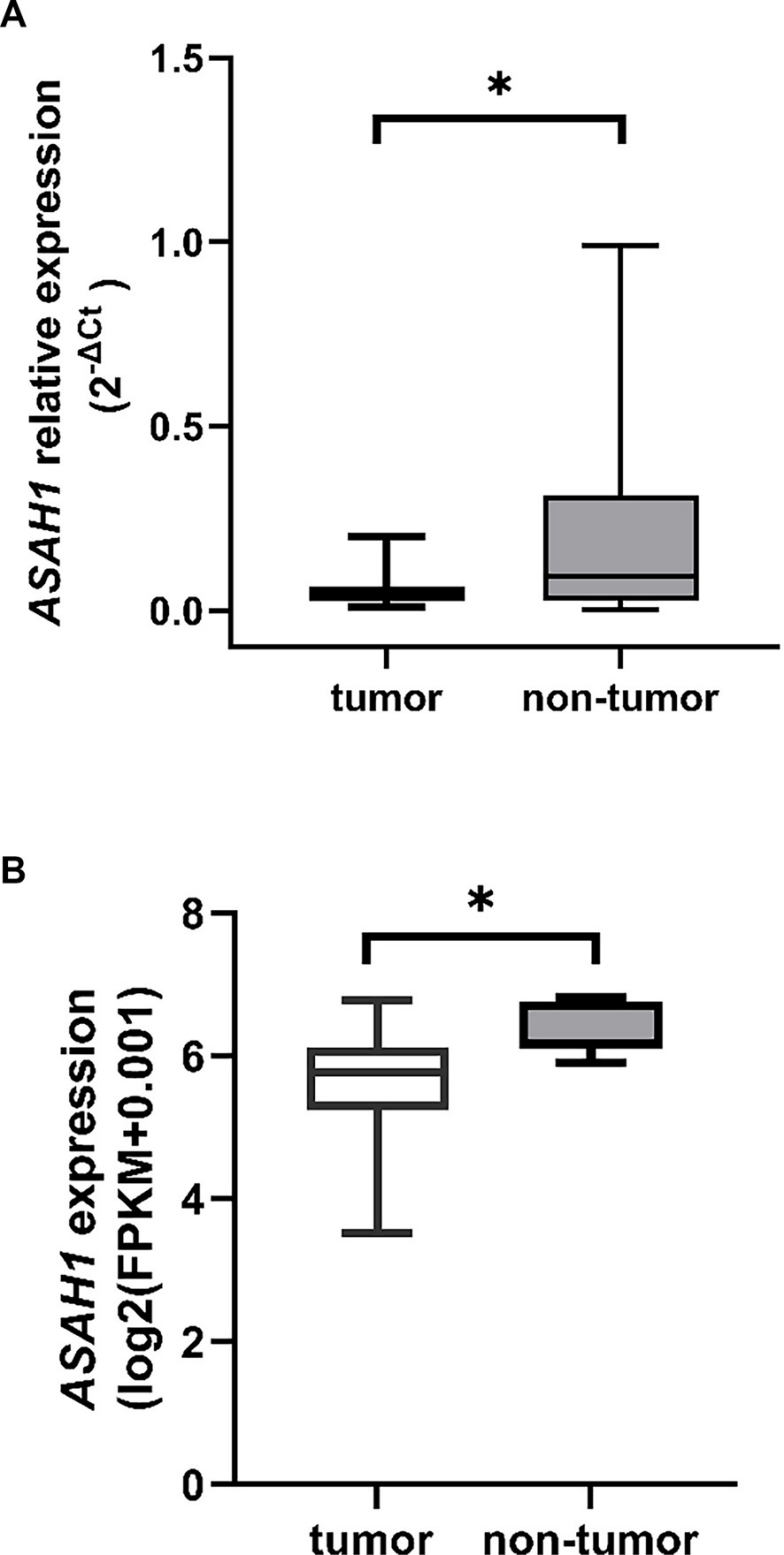
**Analysis of *ASAH1* gene expression in rectal cancer tissue samples.** (A) Relative expression of the *ASAH1* gene in paired tumor (*n* ═ 30) and adjacent non-tumor (*n* ═ 30) tissue samples from patients with locally advanced rectal cancer. Statistical analysis was performed using the Wilcoxon matched-pairs signed-rank test (*P* ═ 0.004). Data are represented as 2^--ΔCt^ values in box plots, illustrating the median, interquartile range, and whiskers indicating minimum and maximum values. (B) *ASAH1* gene expression in tumor (*n* ═ 92) and non-tumor (*n* ═ 10) rectal tissue samples retrieved from The Cancer Genome Atlas Rectum Adenocarcinoma (TCGA-READ) dataset via the UCSC Xena platform. Statistical significance was assessed using the Mann–Whitney *U* test (<0.001). Data are presented as log_2_-transformed FPKM values with an offset of 0.001, displayed as box plots that show the median, interquartile range, and whiskers indicating minimum and maximum values. Abbreviation: *ASAH1*: N–acylsphingosine amidohydrolase 1.

**Figure 2.**
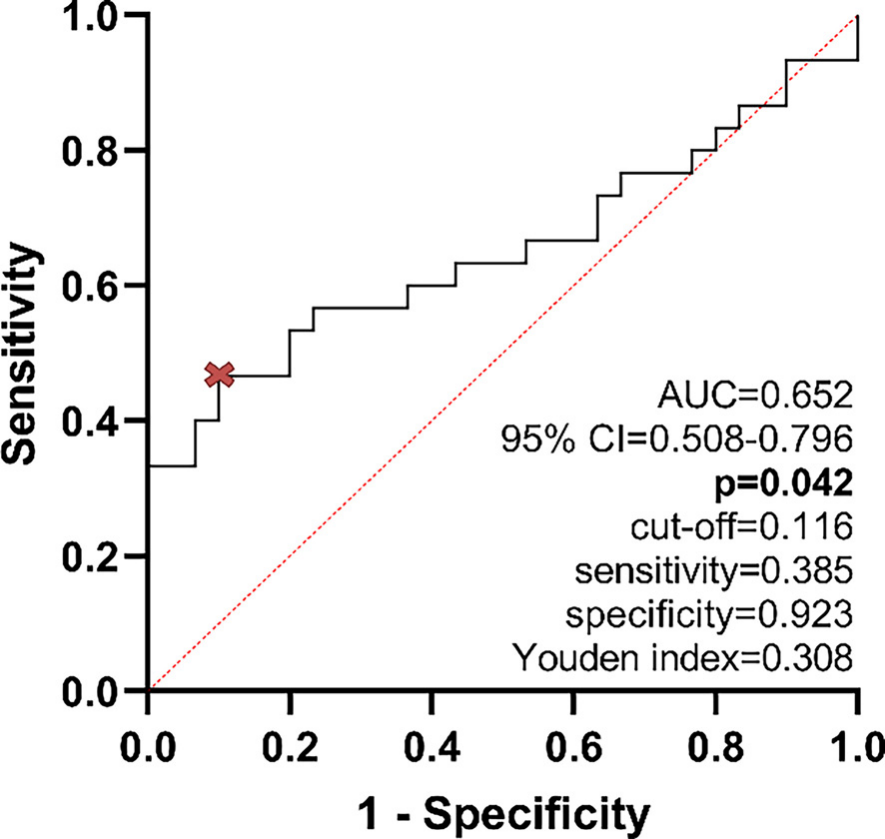
**Receiver operating characteristic (ROC) curve for differentiating tumor and non-tumor rectal tissue based on *ASAH1* gene expression.** The ROC analysis produced an area under the curve (AUC) of 0.652 (95% CI 0.508–0.796, *n* ═ 30, *P* ═ 0.042). The optimal cut-off, indicated by the red symbol, was determined using Youden’s index at 0.116, resulting in a sensitivity of 38.5% and a specificity of 92.3%.

**Table 3:** Correlation of acid ceramidase gene and protein expression with serum markers and tumor apoptotic status in patients with locally advanced rectal cancer (*n* ═ 30)

**Variable**	**CEA (µg/L)**	**CA 19-9 (U/mL)**	**CRP (mg/L)**	**CALLY index**	***BAX/BCL2* ratio**
Tumor tissue *ASAH1* (2^--ΔCt^)	rs = 0.128	rs = 0.215	rs = 0.119	rs ═ −0.164	rs = 0.044
	*P* ═ 0.500 *q* ═ 0.707	*P* ═ 0.253 *q* ═ 0.393	*P* ═ 0.530 *q* ═ 0.707	*P* ═ 0.388 *q* ═ 0.671	*P* ═ 0.816 *q* ═ 0.877
	95% BCa CI --0.262 to 0.488	95% BCa CI --0.167 to 0.589	95% BCa CI --0.291 to 0.491	95% BCa CI --0.496 to 0.207	95% BCa CI --0.367 to 0.453
Serum AC (ng/mL)	rs = 0.013	rs = 0.026	rs = 0.229	rs ═ −0.213	rs ═ −0.536
	*P* ═ 0.945 *q* ═ 0.945	*P* ═ 0.890 *q* ═ 0.890	*P* ═ 0.224 *q* ═ 0.393	*P* ═ 0.259 *q* ═ 0.393	*P* ═ 0.002 *q* ═ 0.021
	95% BCa CI --0.371 to 0.363	95% BCa CI --0.322 to 0.352	95% BCa CI --0.118 to 0.585	95% BCa CI --0.534 to 0.199	95% BCa CI --0.734 to --0.253

Abbreviations: CEA: Carcinoembryonic antigen; CA 19-9: Carbohydrate antigen 19-9; CRP: C-reactive protein; CALLY: CRP-albumin-lymphocyte; *BAX*: BCL2 associated X, apoptosis regulator; *BCL2:* BCL2 apoptosis regulator; *ASAH1*: N–acylsphingosine amidohydrolase 1; AC: Acid ceramidase; rs: Spearman correlation coefficient; BCa CI: Bias-corrected and accelerated bootstrap confidence interval; *P*: Two-tailed *P* value from Spearman correlation; *q*: FDR-adjusted *P* value (Benjamini–Hochberg).

In 70% of patients, the expression of the *ASAH1* gene was significantly reduced in tumor tissues compared to adjacent non-tumor tissues (median reduction of 2.9-fold, IQR 12.6-fold, *n* ═ 21). Conversely, in the remaining 30%, *ASAH1* expression was increased (median increase of 1.9-fold, IQR 3.8-fold, *n* ═ 9). The difference in expression between tumor and adjacent non-tumor tissues was statistically significant across the entire cohort (Wilcoxon matched-pairs signed-rank test, *P* ═ 0.004; Figure 1A). An independent analysis of RNA-seq data from the TCGA-READ cohort, conducted using the UCSC Xena Browser, corroborated a significant decrease in *ASAH1* expression in primary tumor tissues compared to normal rectal tissues (Mann–Whitney *U* test, *P* < 0.001; Figure 1B). ROC analysis indicated a modest ability of *ASAH1* expression to differentiate between tumor and non-tumor tissues (AUC = 0.652, 95% CI 0.508–0.796, *n* ═ 30, *P* ═ 0.042). The optimal cut-off determined by Youden’s index was 0.116, yielding a sensitivity of 38.5% and a specificity of 92.3% (Figure 2).

**Figure S1.**
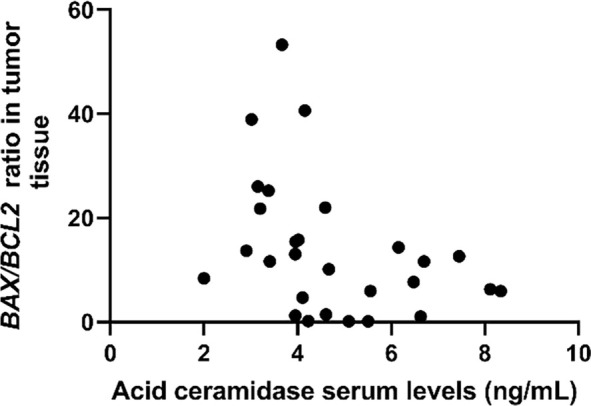
**Correlation between serum acid ceramidase (AC) levels and apoptotic status (*BAX/BCL2* ratio) in tumor tissue.** The *BAX/BCL2* ratio quantifies the expression (2^--ΔCt^) of the pro-apoptotic gene *BAX* relative to the anti-apoptotic gene *BCL2*. The scatter plot illustrates a significant negative correlation between the serum AC levels and the *BAX/BCL2* ratio, assessed using Spearman’s rank correlation (rs ═ --0.536; *n* ═ 30; two-tailed *P* ═ 0.002; *q* ═ 0.021). The *P* value was adjusted using the false discovery rate (FDR) correction according to the Benjamini–Hochberg method. Additionally, the 95% bootstrap confidence interval was calculated, yielding values from --0.734 to --0.253, and the robustness of the correlation was verified through 1000 bootstrap resamples.

Spearman correlation analysis revealed no significant association between *ASAH1* expression in tumor tissue and serum AC levels (rs ═ −0.013, 95% BCa CI --0.324 to 0.263, *n* ═ 30, *P* ═ 0.945, FDR-adjusted *q* ═ 0.945). We further examined the relationships of *ASAH1* expression and serum AC levels with the apoptotic status of tumor tissue (*BAX/BCL2* ratio), as well as with inflammatory markers (CRP and CALLY index) and tumor markers (CEA and CA 19–9) (Table 3). Among the parameters analyzed, only serum AC levels exhibited a significant negative correlation with the *BAX/BCL2* ratio (rs ═ −0.536, 95% BCa CI --0.734 to --0.253, *n* ═ 30, *P* ═ 0.002, FDR-adjusted *q* ═ 0.021; Figure S1).

To assess the relationship between tissue *ASAH1* expression, serum AC levels, and CALLY index with response to nCRT, patients were categorized according to their pathological response: good responders (TRG1+TRG2), moderate responders (TRG3), and poor responders (TRG4+TRG5). However, no significant differences were observed between subgroups for any of the analyzed parameters (Kruskal–Wallis test, *P* > 0.05; Table 4). We evaluated the prognostic potential of these markers concerning overall survival in LARC patients. The median follow-up duration was 62.5 months (IQR 14 months), with 83.3% of patients alive (25/30) at the conclusion of follow-up. In the univariate Cox regression analysis, none of the examined parameters significantly correlated with overall survival (hazard ratios ranging from 1.01–10.05; all *P* > 0.05), with wide confidence intervals reflecting the limited number of observed events (Table S1).

**Table 4:** Association of acid ceramidase at the gene and protein levels, and the CALLY index, with pathological response to neoadjuvant chemoradiotherapy in patients with locally advanced rectal cancer (*n* ═ 26)^#^

**Variable**	**Good responders** **(*n* ═ 5)**	**Moderate responders** **(*n* ═ 9)**	**Poor responders** **(*n* ═ 12)**	***P* value**
Tumor tissue *ASAH1* (2^--ΔCt^), median (IQR)	0.06 (0.08)	0.04 (0.08)	0.04 (0.03)	0.938
Serum AC (ng/mL), median (IQR)	4.2 (1.9)	3.9 (2.8)	4.1 (3.4)	0.451
CALLY index, median (IQR)	3.3 (4.9)	2.9 (7.1)	2.4 (4.0)	0.845

^#^Based on the pathological response to therapy, patients were categorized into three groups: good responders—those exhibiting complete (TRG1) and near-complete (TRG2) tumor regression; moderate responders—those with moderate tumor regression (TRG3); and poor responders—those with minimal tumor regression (TRG4) and no regression (TRG5). Statistical significance among these subgroups was evaluated using the Kruskal–Wallis test. Abbreviations: CALLY: CRP-albumin-lymphocyte; *ASAH1*: N-acylsphingosine amidohydrolase 1; IQR: Interquartile range defined as the difference between the 75th and 25th percentiles; AC: Acid ceramidase.

## Discussion

This exploratory study focused on LARC with the aim of assessing gene and protein expression profiles of AC and exploring their potential clinical relevance, given the limited existing data compared to colon cancer.

We analyzed *ASAH1* gene expression in rectal tissues using two independent approaches: direct quantification via qRT-PCR on samples collected in this study and analysis of publicly available RNA-seq data from the TCGA-READ dataset. Paired tumor and adjacent non-tumor samples were exclusively collected from treatment-naïve stage III LARC patients. Within this cohort, we observed a decrease in *ASAH1* gene expression in tumor tissue, along with a modest ability to differentiate between tumor and non-tumor rectal tissues. A similar decrease was noted in the TCGA-READ dataset, which included transcriptomic data from primary rectal adenocarcinoma samples of stages II–III. This finding aligns with a previous study that combined colon and rectum TCGA datasets to compare the expression of sphingolipid-related genes between tumor and non-tumor tissues, reporting comparable results for the *ASAH1* gene [20]. It should be noted that tumor–normal pairing was not consistently available in the Xena Browser. Nonetheless, despite differences in platforms, methodologies, and potential variations in sample types, similar trends were observed in both analyses, suggesting reduced *ASAH1* expression in tumor tissues. This reduction may reflect functional implications in tumor biology rather than primarily serving as a biomarker for tissue discrimination. Given the scarcity of studies quantifying *ASAH1* expression in human rectal tissues, particularly in non-tumor samples, our findings contribute to a deeper understanding of *ASAH1* in LARC, particularly in the context of limited clinical data.

Based on the *ASAH1* gene expression observed in our study, a corresponding decrease in AC levels might be anticipated, which contrasts with prior reports of elevated AC expression in CRC [21]. These findings indicate that AC is regulated at multiple levels within the tissue, including transcriptional and post-transcriptional mechanisms, and may consequently reflect the differences in enzyme expression observed between the colon and rectum [11]. Since AC levels were not directly measured in tissue samples, we assessed the relationship between serum AC concentrations and *ASAH1* gene expression in tumor tissue, considering gene expression as an indirect indicator of the enzyme. However, no correlation was identified, likely due to the systemic origin of circulating AC. In addition to tumor cells, AC is secreted by the liver, leukocytes, endothelial cells, and other tissues, reflecting an overall rather than tumor-specific sphingolipid metabolism [22–24].

The apoptotic status of tumor tissue is a critical determinant of therapeutic response and may influence patient outcomes. The BAX/BCL2 protein ratio has previously been proposed as a potential indicator of tumor response to adjuvant CRT in rectal cancer patients, underscoring its possible role as a predictive biomarker [25]. In our earlier study, we assessed the *BAX/BCL2* ratio at the gene expression level and found no association with nCRT, providing a complementary molecular perspective. In contrast, long-chain ceramides (C20 CER, C22 CER, and C24 CER) were identified as potential indicators of tumor apoptotic status in patients with LARC [26]. Given that ceramides mediate cell cycle arrest and cell death in response to cellular stress, and that AC is responsible for their degradation, this study investigated the association between *ASAH1* gene expression and serum AC levels with the apoptotic status of tumor tissue. Interestingly, while no significant correlation was observed for *ASAH1* gene expression in tumor tissue, we found a negative correlation between serum AC levels and the *BAX/BCL2* ratio. Reduced serum AC levels and the pro-apoptotic status of tumor tissue suggest that systemic AC measurements could provide insight into tumor apoptotic activity. It is known that lower AC levels may promote apoptosis by allowing ceramide accumulation, as ceramides are not being degraded. Conversely, elevated AC levels not only lead to ceramide degradation but also result in the production of sphingosine, which can be phosphorylated into S1P, a molecule known for its proliferative and pro-survival effects that promote tumor progression [5]. However, further studies are required to confirm this observed relationship. If validated, serum AC could potentially be integrated with tissue-based molecular assessments and other biomarkers for a more comprehensive evaluation.

In our cohort, neither serum AC levels nor *ASAH1* gene expression demonstrated a significant association with treatment response. It is important to note that, in addition to gene and protein expression levels, enzymatic activity is a crucial determinant of AC function. For instance, lower enzyme expression with high activity could yield similar biological consequences as high expression with moderate activity. Therefore, future studies should evaluate both AC expression and activity in serum and rectal tissue, as their combined assessment may provide more meaningful insights into therapeutic response and the potential of AC as a therapeutic target. Moreover, no association between these markers and overall survival was observed. Estimates from Cox regression hazard ratios should be interpreted with caution due to the limited number of events, which reduces statistical power. The absence of significant associations with treatment response or survival may reflect the relatively small sample size, few good responders, high overall survival, and the multifactorial nature of tumor biology, which likely involves complex molecular interactions beyond individual biomarkers. Considering the median follow-up of approximately five years in our cohort, the proportion of patients alive at the end of follow-up (83.3%) is consistent with reported five-year overall survival rates in LARC (81.6%) [27]. Slight differences between studies may reflect variations in patient selection, treatment protocols, or follow-up duration.

To our knowledge, this is the first study to examine the biomarker relevance of the CALLY index in patients with LARC. Although lower preoperative CALLY values have been associated with reduced survival and increased recurrence in CRC, and have shown superior prognostic value compared to other inflammatory markers, we found no association with response to nCRT or overall survival [28–31]. However, examining the CALLY index alongside serum AC levels in future studies could provide a more comprehensive insight into the inflammatory, nutritional, and metabolic status of patients with LARC and their treatment outcomes.

This study focused on LARC samples and, given the relatively small sample size, should be considered exploratory in nature. Nevertheless, the preliminary results offer valuable insights into the potential systemic reflection of local tumor biology and may inform future studies with larger and more representative cohorts. Although neither of the analyzed parameters showed an association with treatment response or survival, the role of sphingolipid metabolism in rectal cancer warrants further investigation, particularly given the limited data available in the current literature. The interplay between ceramide metabolism, apoptosis, inflammation, and nutritional status should be further explored in larger and more diverse patient cohorts.

## Conclusion

The results of this study indicated a significant decrease in *ASAH1* gene expression in rectal tumor tissue compared to adjacent normal mucosa, highlighting the complex regulation of AC in this cancer type. We observed a negative correlation between reduced serum AC levels and the pro-apoptotic status of tumor tissue, suggesting that systemic AC measurements may provide insight into tumor apoptotic activity. However, further research with larger patient cohorts is necessary to confirm these findings and fully elucidate the biomarker relevance of AC in rectal cancer. A deeper understanding of the interplay between ceramide metabolism, inflammation, and tumor progression may enhance patient stratification and guide treatment decisions.

## Supplemental data

**Table S1:** Univariate Cox regression survival analysis for *ASAH1* gene expression, AC levels, and the CALLY index in patients with locally advanced rectal cancer (*n* ═ 30)

**Variable**	**Hazard ratio (95% CI)**	***P* value**
Tumor tissue *ASAH1* (2^--ΔCt^)	10.046 (0.000–65830326.088)	0.773
Serum AC (ng/mL)	1.098 (0.652–1.849)	0.923
CALLY index	1.013 (0.785–1.306)	0.726

Abbreviations: ASAH1: N-acylsphingosine amidohydrolase 1; AC: Acid ceramidase; CALLY: CRP-albumin-lymphocyte.

## Data Availability

Data are available from the corresponding author upon reasonable request.
